# Dietary saturated fat and cholesterol: cracking the myths around eggs and cardiovascular disease

**DOI:** 10.1017/jns.2023.82

**Published:** 2023-09-11

**Authors:** Rona Antoni

**Affiliations:** Department of Nutrition, Food and Exercise Sciences at University of Surrey, Guildford GU2 7WG, UK

**Keywords:** Cardiovascular disease, Cholesterol, Eggs, Saturated fat

## Abstract

Whilst dietary cholesterol guidelines have waivered through the years with historic restrictions lifted for the majority of the general population, recommendations to reduce saturated fat intake have been the mainstay of dietary guidelines since the 1980s and were recently reinforced by the Scientific Advisory Committee on Nutrition (SACN). Cholesterol metabolism is complex, with saturated fat known to have a more significant contribution at raising levels of low-density lipoprotein (LDL) cholesterol, a well-established risk factor for cardiovascular disease (CVD). However, it is clear from metabolic research that hyper-responsiveness to both dietary cholesterol and saturated fat exists; hence, for specific subsets of the population, reductions in both nutrients may be indicated. With this in mind, the current article aims to provide an overview of the mechanisms underlying biological variation in responsiveness and introduces research currently underway which will hopefully identify simple biomarkers that can be used to predict responsiveness and permit tailored, personalised, dietary advice. Eggs are a well-known source of dietary cholesterol whilst being low in saturated fat. A common question encountered in clinical practice is must individuals limit intake to manage blood cholesterol levels. This article summarises key recent papers which confirm that eggs can be enjoyed as part of a healthy balanced diet, whilst highlighting the need for further research in certain population groups, e.g. in individuals with diabetes.

## Dietary saturated fat and cholesterol: cracking the myths around eggs and cardiovascular disease

Heart and circulatory diseases including stroke and atherosclerotic cardiovascular diseases (CVD) account for approximately a quarter of deaths in the UK. Moreover, there are 7⋅6 million people living with heart and circulatory diseases in the UK, a trend that is likely to rise in the context of our increasingly aging population and improved survival rates following events. Thus, focusing on population lifestyle measures that can reduce morbidity and mortality from heart and circulatory diseases is a key priority for the health and wealth of the nation.^(1)^

High blood cholesterol, or more specifically low-density lipoprotein (LDL) cholesterol, is a significant risk factor for developing heart and circulatory diseases, attributed to one in four associated deaths in the UK.^(1)^ Diet is a key factor influencing circulating levels of blood cholesterol, with the latest research findings on the impact of dietary cholesterol and saturated fat being the main focus of the current article.

### Dietary cholesterol: hepatic and intestinal regulation, hyper- and hypo-cholesterol absorbers

The major dietary sources of cholesterol include eggs, offal, dairy food, and shellfish. The average cholesterol intake for individuals consuming a typical Westernised diet is approximately 300–400 mg/d, whilst bile contributes an additional 1000 mg/d. On average, 56 % of this cholesterol is absorbed but is the subject of significant individual variation, ranging between 29 and 80 %.^(2)^ One such determinant of this variation is a receptor located in enterocytes called the Niemann–Pick C1-like (NPC1L1) receptor, responsible for the uptake of cholesterol (and phytosterols) from the gut lumen. Genetic variation exists in the NPC1L1 receptor, with many variants resulting in decreased activity of this receptor.^(3)^

Cholesterol homeostasis is controlled mainly by endogenous synthesis, intestinal absorption, and hepatic excretion. When gut absorption of cholesterol is enhanced, e.g. due to increased dietary intake, hepatic concentrations are subsequently increased. To protect against lipotoxicity (due to excess free cholesterol in the cell), the liver responds in a number of ways: (1) by reducing cholesterol synthesis; (2) by reducing cholesterol absorption by downregulating the NPC1L1 receptor; (3) by increasing hepatobiliary cholesterol excretion into bile; and (4) by reducing LDL receptor expression, therefore reducing hepatic uptake of LDL cholesterol and increasing circulating levels. This interplay between the liver and intestine acts to mitigate any increase in dietary cholesterol intake; as such, evidence from observational studies generally does not indicate a significant association between dietary cholesterol with cardiovascular disease (CVD) risk.^(4)^ However, it is important to note that hyper-absorbers of dietary cholesterol have a higher incidence of atherosclerotic CVD, and as such benefit from significantly reducing total cholesterol in the diet to reduce circulating levels of LDL cholesterol, a well-established risk factor for CVD.^(5)^

### Saturated fat: effects on LDL cholesterol

Saturated fat is found predominantly in animal products, such as meat, butter, ghee, lard, and dairy products including cheese and yogurt. Products made using these types of fats, such as cakes, biscuits, and pastries, can also be high in saturated fat, as well as some vegetable fats including cocoa butter, palm, and coconut oil. The average UK intake of saturated fat intake for men and women is 12⋅3 % of total energy based on NDNS data. Saturated fats increase LDL cholesterol by inhibiting the activity of the LDL receptor (therefore increasing circulating levels) and upregulating the synthesis of LDL particles.^(6)^ Tightly controlled metabolic ward studies from the early 1950s were among the first to show that dietary saturated fats, when compared to unsaturated fatty acids, significantly increase blood cholesterol levels.^(7,8)^ LDL particles comprise a heterogeneous group of particles varying in size and density. A cross-over study by Bergeron *et al.*^(9)^ demonstrated that the observed increase in serum cholesterol from high saturated fat diets stems from an increase in the concentration of large LDL particles, whereas small- and medium-sized LDL particles were unaffected. Small- and medium-density LDL are considered more atherogenic due to decreased hepatic clearance by the LDL receptor and enhanced anchoring to LDL receptor-independent binding sites in extrahepatic tissues including the arterial wall.^(10)^ Anecdotally, the atherogenicity of high saturated fat diets has been questioned due to the fact they increase concentrations of larger ‘less atherogenic’ LDL subclasses. It should, however, that high concentrations of large LDL remain atherogenic especially when accompanied by an increase in LDL particle number as it indicates a greater number of particles available to penetrate the arterial wall, leading to the formation of plaques and atherosclerosis.^(11)^ Indeed, in the aforementioned cross-over study,^(9)^ an increase in apolipoprotein B (a surrogate marker for LDL particle number) was also observed in conjunction with the rise in LDL cholesterol in response to high saturated fat diets, highlighting an important mechanism in which high saturated fat diets can increase cardiovascular risk.

### Saturated fat and CVD risk

One of the most comprehensive meta-analyses to date of randomised controlled trials, following gold standard Cochrane principles, demonstrated a 21 % reduction in combined cardiovascular events when saturated fats were reduced for at least 2 years.^(12)^ This cardioprotective effect is likely in part due to observed reductions in LDL cholesterol. The study also explored the impact of macronutrient substitution and showed that replacing saturated fat with polyunsaturated fats appeared more protective of cardiovascular events than replacing with carbohydrates. Further evidence from a group of four randomised controlled trials also found benefits of replacing saturated fats with polyunsaturated fats, with a significant (30 %) reduction in coronary heart disease reported (RR: 0⋅71; 95 % CI: 0⋅61, 0⋅83).^(13)^

### Negative research: sources of confounding

It should be noted that meta-analyses have not been unanimous in this finding, however, with several failing to demonstrate an association between dietary saturated fat and CVD risk.^(14,15)^ As such, the subject of saturated fats and their links to CVD risk remains a hotly debated area. Meta-analyses which have failed to find an association often fail to consider nutrient substitution and are confounded further still by factors such as food matrix effects influencing dietary saturated fat absorption and co-existence of other cardioprotective compounds in foods. In the case of dairy for instance, inverse association with full-fat dairy including fermented products and cardiometabolic health outcomes including CVD disease have been shown.^(16)^ Moreover, yogurt and cheese consumption generally does not seem to exert detrimental effects on blood lipids as would be predicted by the saturated fat content, likely due to food matrix effects. In fact, in one controlled-feeding study conducted by Brassard *et al.*,^(17)^ LDL cholesterol was significantly lower (−3⋅3 %) after consumption of a diet matched for saturated fat from cheese compared with butter.

### Variability in LDL cholesterol responses to saturated fat

Recent studies have also noted the phenomenon of inter-individual variation in LDL cholesterol response to saturated fat. For example, one study showed that an increase in saturated fat of 6⋅1 % total energy produced variation in LDL cholesterol ranging between +45 and −20 %.^(18)^ As summarised by Griffin *et al.*,^(19)^ a number of mechanisms can be at play including but not limited to: (1) genetics, e.g. apolipoprotein E (protein located on LDL particles) phenotype which influences the affinity of LDL cholesterol binding to its receptor; (2) bile acids — the composition can influence fat absorption and bile acids can also influence gene expression by acting as ligands for nuclear receptors that regulate the transcription of lipid metabolism genes; and (3) the composition of gut microbiota — which influences bile acid composition and gut permeability. Gut microbiota are also a source of short-chain fatty acids including acetate and propionate, which have been shown to stimulate and inhibit cholesterol biosynthesis, respectively.

To further elucidate the above mechanism, research is currently underway via a BBSRC-funded multi-centre project undertaken by the Universities of Reading, Surrey and Imperial College London in healthy individuals (https://gtr.ukri.org/projects?ref = BB%2FP009891 %2F1). Furthermore, the study aims to apply metabolomic techniques to identify serum and urinary biomarkers of these metabolic differences that can be used to predict an individual's responsiveness to saturated fat.

## Dietary guidelines for saturated fat

Saturated fat reduction has been the mainstay of dietary guidelines since the 1980s. Despite recent contentious debate between health professionals, journalists, and the general public, two independent reports by SACN and WHO aligned in their recommendations that total dietary saturated fat should be limited.^(20)^ In the UK, it is currently recommended that intakes do not exceed 10 % of total energy and should ideally be substituted by unsaturated fatty acids, although one could argue that later iterations of guidelines should consider emerging research particularly around dairy foods and focus on food patterns over absolute reductions in saturated fat.

## Dietary guidelines for cholesterol

In part due to the recognition of the greater relative impact of saturated fats on LDL cholesterol, dietary guidelines have slowly shifted away from recommending absolute limits on cholesterol intake. Organisations such as the British Heart Foundation and Heart UK do not recommend absolute restrictions but recommend lower saturated fat options such as eggs, shellfish, and offal. It should be acknowledged that NICE guidelines (CG181) recommend lifestyle modification including saturated fat and restriction of dietary cholesterol to <300 mg/d for those at high risk of/having CVD, and less than 200 mg for those with familial hypercholesterolaemia (FH). The 2019 European Society of Cardiology (ESC) and European Atherosclerosis Society (EAS) guidelines for the management of dyslipidaemia advocate moderate consumption of eggs for the management of dyslipidaemia. In quantitative terms, Heart UK advises even individuals in high-risk groups can eat three or four eggs a week, and shellfish such as prawns up to once or twice a week.

## Eggs and CVD

### Positive research

Eggs are a well-known source of dietary cholesterol, and therefore received a lot of bad press in the 1960s and 1970s owed to purported harmful effects of excess consumption. The average medium egg contains 177 mg of cholesterol but only 1⋅3 g of saturated fat. As reviewed by Griffin,^(21)^ the general consensus after 60 years of research is that dietary cholesterol, chiefly from eggs, exerts a relatively small effect on serum LDL cholesterol and CVD risk, in comparison with other diet and lifestyle factors. More recently, a study of more than 17 700 people from 50 countries confirmed a lack of significant relationship with increased blood lipids, blood pressure, or deaths from CVD.^(22)^ Another recent example is a systematic review and meta-analysis by Krittanawong *et al.*^(23)^ The objective of this review was to explore the association between egg consumption and CVD, including prospective or cross-sectional studies, using a random effects model. The meta-analysis identified 23 prospective studies with a median follow-up of 12⋅28 years. A total of 1 415 839 individuals with a total of 123 660 cases and 157 324 CVD events across an array of different countries. The study failed to find a significant association between egg consumption and increased risk of overall CVD events (HR 0⋅99; 95 % CI, 0⋅93–1⋅06; I2 = 72⋅1 %). Furthermore, compared with the consumption of 0–1 eggs/d, higher egg consumption (>1 egg/d) was associated with a significantly decreased risk of coronary arterydisease (HR 0⋅89; 95 %CI, 0⋅86–0⋅93; I2 = 0 %).

### Negative research: critical overview of limitations

Nevertheless, it should be noted that a small but not insignificant number of studies have highlighted the possibility for negative health consequences associated with increased egg consumption, although in many of these studies, collection of dietary data does not accurately record egg consumption nor the way eggs are cooked. Thus, egg consumption may be linked to an unhealthy diet. For example, recently a study by Ruggiero *et al.*^(24)^ highlighted associations between egg consumption and risk of all-cause and CVD mortality. The study recruited 24 325 men and women from Italy aged >=35 years (average 54 years) followed up for 8⋅3 years, with a mean consumption of 1⋅8 eggs/week and a mean dietary cholesterol intake of 322 mg/d. In a multivariable-adjusted model including the Mediterranean diet score (MDS), as compared to participants reporting lower egg consumption, consuming more than 4 eggs/week was associated with increased risk of all-cause (HR = 1⋅50; 95 %CI 1⋅13–1⋅99), CVD (HR = 1⋅75; 1⋅07–2⋅87) and cancer mortality (HR = 1⋅52; 0⋅99–2⋅33). The region's recommended intake of 2–4 eggs/week also led to an increased all-cause (HR = 1⋅22; 1⋅01–1⋅46) and CVD mortality risk (HR = 1⋅43; 1⋅03–1⋅97). Furthermore, each additional egg per week was associated with a higher risk of all-cause (HR = 1⋅06; 1⋅00–1⋅12) and CVD mortality (HR = 1⋅10; 1⋅01–1⋅21). Limitations of this study include the fact that the study is a longitudinal epidemiological analysis, and therefore can only show associations and not causation and are vulnerable to residual confounding or confounding by unmeasured factors. The CIs for certain outcomes (e.g. CVD mortality) were wide and bordering on 1. The study only recorded a small number of total deaths (*n* 838) over a relatively short period of follow-up, with fewer still falling in the highest egg consumption category. They also used semi-quantitative food frequency questionnaires based on intake over the last 12 months, and are therefore limited as they do not capture life-course changes. The authors reported that the preferred cooking methods for eggs were unknown. Dietary cholesterol explained 43 and 39 % of the association of eggs with all-cause and CVD mortality; however, egg consumption only contributed to 14⋅6 % of total cholesterol intake in the diet in this study. Participants with a greater egg intake (>4 eggs/week) tended to have a lower baseline MDS and consumed less fruit, nuts, cereals, and more meat and meat products. The contribution of protein and fat to total energy intake increased across the four categories of egg consumption, whereas the contribution of fibre intake decreased as egg consumption increased. Although the statistical model used accounted for MDS (index of dietary quality), controlling for confounding through statistical means is imperfect and there is potential for residual confounding.

Another recent meta-analysis of 17 studies of RCTs published between 1984 and 2017 used random effects to explore the association of egg consumption on LDL and HDL concentrations.^(25)^ Eligible trials only investigated healthy subjects. The study compared a ‘more egg consumption’ (MEC) group where egg intakes ranged from 1 to 3 eggs/d, or 1 extra egg. The intervention period for these studies ranged from 21 to 84 d. Overall, it was found that the MEC group exhibited a significant elevation in the LDL cholesterol/HDL-cholesterol ratio than did control groups (MD = 0⋅14; 95 % CI: 0⋅05–0⋅22; I2 = 25 %), which was driven by an increase in LDL cholesterol with no statistically significant effect on HDL-cholesterol. With one and three or more egg consumption, the MEC group did not have a significant difference from the control group, whilst two egg consumption showed a significantly higher LDL cholesterol/HDL-cholesterol ratio (MD = 0⋅13; 95 % CI: 0⋅01–0⋅26; I2 = 13 %). Limitations of this study, however, included the short study durations of less than a year, and the authors conceded that they were unable to determine the foods accompanying the eggs and differing background diets could have influenced the results. The study looked at LDL cholesterol/HDL-cholesterol but no end point data are available. Furthermore, the control diets were heterogeneous — ranging from no egg, regular diet, oatmeal to egg white. It is also strange that statistical significance was observed with 2-egg consumption but not with 1 or >3.

In summary, whilst some studies suggest negative associations between egg consumption and CVD risk, common shortcomings among these studies include inaccurate methods of collecting dietary information, a failure to consider egg cooking methods (e.g. frying) and co-consumption of other cholesterol-containing foods linked to CVD risk (e.g. processed meats) which may confound observations. Indeed, the British Heart Foundation recommends that ‘*for most people, the way you cook eggs or the foods you serve alongside are more important than the eggs themselves. Poaching or boiling is healthier than frying in bacon fat or scrambling with butter.’*

### Eggs and diabetes

Some epidemiological studies have suggested that there may be an increased risk of CVD in individuals with diabetes who consume 7 or more per week.^(21)^ For example, one subgroup analysis by Qureshi *et al.*,^(26)^ consuming more than 6 eggs/week was associated with an increased risk of CHD (RR 2⋅0, 95 % CI 1⋅0–3⋅8). Another prospective cohort study by Djousse & Gaziano^(27)^ also noted an increase in mortality with increasing egg consumption when comparing individuals consuming <1 egg/d versus 7 or more (HR 2⋅01 95 % CI: 1⋅26, 3⋅20), with the authors speculating that among subjects with diabetes, dietary cholesterol may encourage a less favourable lipoprotein profile. However, the authors conceded that the study could not examine the effects of saturated fat, markers of insulin resistance, lipids, or other nutrients on the observed observations. Most recently, a meta-analysis by Djousse *et al.*^(28)^ explored the link between egg consumption and risk of developing type 2 diabetes in a pooled analysis of nine US cohorts. The authors observed that previous meta-analyses demonstrating a link to type 2 diabetes were limited to US cohorts, with no such link observed in non-US studies, a finding replicated by Drouin-Chartier *et al.*^(29)^ A particular strength of the Djousse *et al.*^(28)^ meta-analysis included large sample size with an adequate number of incident type 2 diabetes for sub-analyses and use of standardised protocols for all cohorts. Moreover, to control for confounding by modifiable factors inherent to US populations, this meta-analysis adjusted for dietary patterns and demonstrated consumption of more than 7 eggs/week was associated with a 27 % (95 % CI: 16 %, 37 %) elevated risk of type 2 diabetes when compared to those who consumed none. No clear biological mechanism could be identified, however again, the strength of association was greatest in subjects with poorer diet. The authors acknowledged that unmeasured and/or residual confounding, e.g. cooking methods may explain the observed findings. In the US in general, the habit of eating processed meats with eggs is not always accounted for models used to examine the associations.

Ultimately, most evidence suggesting an increased risk of CVD and type 2 diabetes from egg consumption in individuals with diabetes stems from secondary analyses and is largely observational, i.e. hypothesis generating and thus requires further focused clinical trials. Patients with diabetes are heterogeneous, and response to egg consumption may differ by type of diabetes.

More broadly speaking, eggs also contain other beneficial nutrients, e.g. protein, B vitamins, vitamin D, selenium, and iodine. Eggs are low energy, high protein, and therefore particularly beneficial for weight-reducers; in populations including those with diabetes, increased egg consumption (12 × /week *v.* <2 × /week) in the context of a hypo-energetic, low saturated fat diet has been shown to promote weight-loss with no adverse effects on blood lipids or markers of glycaemic control.^(30)^

## Conclusions

In summary, two things are clear — individual responsiveness to dietary cholesterol varies person to person. Secondly, saturated fat which often co-exists in foods containing cholesterol is the more dominant driver of LDL cholesterol and can be modulated by factors such as the food matrix as shown for dairy foods. Nonetheless, it is prudent to explore the factors that determine an individual's responsiveness to dietary fat — both saturated fat and cholesterol — and determine predictive variables that can be used to provide tailored nutritional advice to individuals such as blood biomarkers. This research is currently underway. Eggs are not a large source of dietary saturated fat with butter and full-fat dairy products being the main vehicles in the typical British diet. Negative research linking egg consumption and CVD risk exists but is subject to numerous limitations associated with epidemiological research. Within the general population, eggs can be enjoyed as part of a healthy balanced diet (with historic restrictions on consumption levels removed in more recent iterations of dietary guidelines) and can be beneficial for weight-loss as well as a cheap nutritious source of protein. Restrictions on total dietary cholesterol as a whole are indicated only in very specific high-risk groups, e.g. hyper-absorbers and those with FH whilst more research is required in individuals with diabetes.

## Abbreviations

CI, Confidence interval
CVD, Cardiovascular disease
EAS, European Atherosclerosis Society
ESC, European Society of Cardiology
FH, Familial hypercholesterolaemia
HR, Hazard ratio
HDL: High-density lipoprotein
LDL, Low-density lipoprotein
NDNS, National Diet and Nutrition Survey
NICE, National Institute of Clinical Excellence
NPC1L1, Niemann-Pick C1-like
RR, Relative risk
SACN, Scientific Advisory Committee on Nutrition
WHO, World Health Organisation

## References

[1] British Heart Foundation. CVD Statistics UK Factsheet. 2022. Accessed July 23, 2023. https://fdocuments.in/document/cvd-statistics-bhf-uk-factsheet-statistics-bhf-uk-factsheet-last-reviewed.html?page=2.

[2] Schade DS, Gonzales K, Kaminsky N, Adolphe A, Shey L & Eaton RP. Resolving the egg and cholesterol intake controversy: New clinical insights into cholesterol regulation by the liver and intestine. Endocrinol Pract. 2022;28(1):102–109. 10.1016/j.eprac.2021.09.004 34547473

[3] Fahmi S, Yang C, Esmail S, Hobbs HH & Cohen JC. Functional characterization of genetic variants in NPC1L1 supports the sequencing extremes strategy to identify complex trait genes. Hum Mol Genet. 2008;17(14):2101–2107. 18413323 10.1093/hmg/ddn108 PMC2441721

[4] Carson JAS, Lichtenstein AH, Anderson CAM, et al. Dietary cholesterol and cardiovascular risk: A science advisory from the American Heart Association. Circulation. 2020;141(3):e39–e53. 31838890 10.1161/CIR.0000000000000743

[5] Silbernagel G, Chapman JM, Genser B, et al. High intestinal cholesterol absorption is associated with cardiovascular disease and risk alleles in ABCG8 and ABO: Evidence from the LURIC and YFS cohorts and from a meta-analysis. J Am Coll Cardiol. 2013;62(4):291–299. 23707316 10.1016/j.jacc.2013.01.100

[6] Dietschy JM. Dietary fatty acids and the regulation of plasma low density lipoprotein cholesterol concentrations. J Nutr. 1998;128(2 Suppl):444S–448S. 9478045 10.1093/jn/128.2.444S

[7] Kinsell LW, Partridge J, Boling L, Margen S & Michaels G. Dietary modification of serum cholesterol and phospholipid levels. J Clin Endocrinol Metab. 1952;12:909–913. 14938428 10.1210/jcem-12-7-909

[8] Ahrens EH, Blankenhorn DH & Tsaltas TT. Effect on human serum lipids of substituting plant for animal fat in diet. Soc Exp Biol Med. 1954;86:872–878. 10.3181/00379727-86-21260 13204382

[9] Bergeron N, Chiu S, Williams PT, King SM & Krauss RM. Effects of red meat, white meat, and nonmeat protein sources on atherogenic lipoprotein measures in the context of low compared with high saturated fat intake: A randomized controlled trial. Am J Clin Nutr. 2019;110(1):24–33. 31161217 10.1093/ajcn/nqz035 PMC6599736

[10] Galeano NF, Al-Haideri M, Keyserman F, Rumsey SD & Decklebaum RJ. Small dense low density lipoprotein has increased affinity for LDL receptor-independent cell surface binding sites: A potential mechanism for increased atherogenicity. J Lipid Res. 1998;39(6):1263–1273. 9643358

[11] Pencina KM, Pencina MJ, Lawler PR, et al. Interplay of atherogenic particle number and particle size and the risk of coronary heart disease. Clin Chem. 2023;69(1):48–55. 36331823 10.1093/clinchem/hvac172 PMC10833272

[12] Hooper L, Martin N, Jimoh OF, Kirk C, Foster E & Abdelhamid AS. Reduction in saturated fat intake for cardiovascular disease. Cochrane Database Syst Rev. 2020;5:CD011737. 32428300 10.1002/14651858.CD011737.pub2 PMC7388853

[13] Sacks FM, Lichtenstein AH, Wu JHY, et al. Dietary fats and cardiovascular disease: A presidential advisory from the American Heart Association. Circulation. 2017;136:e1–23. 28620111 10.1161/CIR.0000000000000510

[14] Siri-Tarino PW S, Hu FB & Krauss RM. Meta-analysis of prospective cohort studies evaluating the association of saturated fat with cardiovascular disease. Am J Clin Nutr. 2010;91:535–546. 20071648 10.3945/ajcn.2009.27725 PMC2824152

[15] Harcombe Z, Baker JS & Davies B. Evidence from prospective cohort studies does not support current dietary fat guidelines: A systematic review and meta-analysis. Br J Sports Med. 2017;51:1743–1749. 27697938 10.1136/bjsports-2016-096550

[16] Hirahatake KM, Astrup A, Hill JO, Slavin JL, Allison DB & Maki KC. Potential cardiometabolic health benefits of full-fat dairy: The evidence base. Adva Nutr. 2020;11(3):533–547. 10.1093/advances/nmz132 PMC7231591 31904812

[17] Brassard D, Tessier-Grenier M, Allaire J, et al. Comparison of the impact of SFAs from cheese and butter on cardiometabolic risk factors: A randomized controlled trial. Am J Clin Res. 2017;105(4):800–809. 10.3945/ajcn.116.150300 28251937

[18] Vafeiadou K, Weech M, Altowaijri H, et al. Replacement of saturated with unsaturated fats had no impact on vascular function but beneficial effects on lipid biomarkers, E-selectin, and blood pressure: Results from the randomized, controlled dietary intervention and VAScular function (DIVAS) study. Am J Clin Nutr. 2015;102:40–48. 26016869 10.3945/ajcn.114.097089

[19] Griffin BA, Mensink RP & Lovegrove JA. Does variation in serum LDL-cholesterol response to dietary fatty acids help explain the controversy over fat quality and cardiovascular disease risk? Atherosclerosis. 2021;328:108–113. 33863548 10.1016/j.atherosclerosis.2021.03.024

[20] Antoni RC & Griffin BA. Draft reports from the UK's scientific advisory committee on nutrition and world health organization concur in endorsing the dietary guideline to restrict intake of saturated fat. Nutr Bull. 2018;43:206–2011.

[21] Griffin BA. Eggs: Good or bad? Proc Nutr Soc. 2016;75(3):259–264. 27126575 10.1017/S0029665116000215

[22] Dehghan M, Mente A, Rangarajan S, et al. Association of egg intake with blood lipids, cardiovascular disease, and mortality in 177,000 people in 50 countries. Am J Clin Nutr. 2020;111:795–803. 31965140 10.1093/ajcn/nqz348 PMC7138651

[23] Krittanawong C, Narasimad B, Wang Z, et al. Association between egg consumption and risk of cardiovascular outcomes: A systematic review and meta-analysis. Am J Med. 2020;134(1):76–83. 32653422 10.1016/j.amjmed.2020.05.046

[24] Ruggiero E, Castelnuovo AD, Costanzo S, et al. Egg consumption and risk of all-cause and cause-specific mortality in an Italian adult population. Eur J Nutr. 2021;60:3691–3702. 33763719 10.1007/s00394-021-02536-w PMC8437843

[25] Li MY, Chen JH, Chen C & Kang YN. Association between Egg consumption and cholesterol concentration: A systematic review and meta-analysis of randomized controlled trials. Nutrients. 2020;12(7):1995. 32635569 10.3390/nu12071995 PMC7400894

[26] Qureshi AI, Suri FK, Ahmed S, Nasar A, Divani AA & Kirmani JF. Regular egg consumption does not increase the risk of stroke and cardiovascular diseases. Med Sci Monitor. 2007;13(1):Cr1-8. 17179903

[27] Djousse L & Gaziano JM. Egg consumption in relation to cardiovascular disease and mortality: The physicians’ health study. Am J Clin Nutr. 2008;87(4):964–969. 18400720 10.1093/ajcn/87.4.964 PMC2386667

[28] Djousse L, Zhou G, McClelland RL, et al. Egg consumption, overall diet quality, and risk of type 2 diabetes and coronary heart disease: A pooling project of US prospective cohorts. Clin Nutr. 2021;40:2475–2482. 33932789 10.1016/j.clnu.2021.03.003 PMC8564713

[29] Drouin-Chartier JP, Schwab AL, Chen S, et al. Egg consumption and risk of type 2 diabetes: Findings from 3 large US cohort studies of men and women and a systematic review and meta-analysis of prospective cohort studies. Am J Clin Nutr. 2020;112(3):619–630. 32453379 10.1093/ajcn/nqaa115 PMC7458776

[30] Fuller NR, Caterson ID, Sainsbury A, et al. The effect of a high-egg diet on cardiovascular risk factors in people with type 2 diabetes: The diabetes and egg (DIABEGG) study-a 3-mo randomized controlled trial. Am J Clin Nutr. 2015;101(4):705–713. 25833969 10.3945/ajcn.114.096925

